# Mechanisms of gene rearrangement in 13 bothids based on comparison with a newly completed mitogenome of the threespot flounder, *Grammatobothus polyophthalmus* (Pleuronectiformes: Bothidae)

**DOI:** 10.1186/s12864-019-6128-9

**Published:** 2019-10-30

**Authors:** Hairong Luo, Xiaoyu Kong, Shixi Chen, Wei Shi

**Affiliations:** 1 CAS Key Laboratory of Tropical Marine Bio-resources and Ecology, South China Sea Institute of Oceanology, Guangzhou, 510301 China; 2 University of Chinese Academy of Sciences, Beijing, 100049 China

**Keywords:** Flatfish, Bothidae, Mitogenome, Rearrangement mechanism, Dimeric mitogenomes, Intergenic spacer

## Abstract

**Background:**

The mitogenomes of 12 teleost fish of the bothid family (order Pleuronectiformes) indicated that the genomic-scale rearrangements characterized in previous work. A novel mechanism of genomic rearrangement called the Dimer-Mitogenome and Non-Random Loss (DMNL) model was used to account for the rearrangement found in one of these bothids, *Crossorhombus azureus*.

**Results:**

The 18,170 bp mitogenome of *G. polyophthalmus* contains 37 genes, two control regions (CRs), and the origin of replication of the L-strand (O_L_). This mitogenome is characterized by genomic-scale rearrangements: genes located on the L-strand are grouped in an 8-gene cluster (*Q*-*A*-*C*-*Y*-*S*_*1*_-*ND6*-*E*-*P*) that does not include *tRNA*-*N*; genes found on the H-strand are grouped together (*F*-*12S* … *CytB*-*T*) except for *tRNA*-*D* that was translocated inside the 8-gene L-strand cluster. Compared to non-rearranged mitogenomes of teleost fishes, gene organization in the mitogenome of *G. polyophthalmus* and in that of the other 12 bothids characterized thus far is very similar. These rearrangements could be sorted into four types (Type I, II, III and IV), differing in the particular combination of the CR, *tRNA*-*D* gene and 8-gene cluster and the shuffling of *tRNA*-*V*. The DMNL model was used to account for all but one gene rearrangement found in all 13 bothid mitogenomes. Translocation of *tRNA*-*D* most likely occurred after the DMNL process in 10 bothid mitogenomes and could have occurred either before or after DMNL in the three other species. During the DMNL process, the *tRNA*-*N* gene was retained rather than the expected *tRNA*-*N′* gene. *tRNA*-*N* appears to assist in or act as O_L_ function when the O_L_ secondary structure could not be formed from intergenic sequences. A striking finding was that each of the non-transcribed genes has degenerated to a shorter intergenic spacer during the DMNL process. These findings highlight a rare phenomenon in teleost fish.

**Conclusions:**

This result provides significant evidence to support the existence of dynamic dimeric mitogenomes and the DMNL model as the mechanism of gene rearrangement in bothid mitogenomes, which not only promotes the understanding of mitogenome structural diversity, but also sheds light on mechanisms of mitochondrial genome rearrangement and replication.

## Background

The mitogenome of most fish contains 37 genes, including 13 protein-coding genes, two ribosomal RNA (rRNA), and 22 transfer RNA (tRNA) genes. Most of these genes are located on the heavy strand (H-strand), only *ND6* and eight tRNA genes (*N*, *Q*, *A*, *C*, *Y*, *S*_*1*_, *E* and *P*) are located on the light strand (L-strand). Additionally, the mitogenome contains two non-coding regions; the origin of replication of the L-strand (O_L_) and the control region (CR). The CR includes the origin of replication of the H-strand (O_H_) as well as the transcription initiation site for both the L-and H-strands [1–3]. Three types of gene rearrangements have been observed in the mitogenomes of animals: shuffling, translocation, and inversion [4–7]. Before gene inversion was discovered in tongue fish [8], only gene shuffling and translocation had been reported in fishes [9–11]. Since then, an increasing number of rearranged mitogenomes of flatfishes featuring all three gene rearrangement types have been found [12–16]. One such representative case was the mitochondrial gene rearrangement in the blue flounder, *Crossorhombus azureus* [14]. In this mitogenome, genes were grouped with identical transcriptional polarities, including a cluster of eight genes on the L-strand (8-gene cluster, *Q*-*A*-*C*-*Y*-*S*_*1*_-*ND6*-*E*-*P*) that did not include *tRNA*-*N*, and a cluster of genes (*F*-*12S* … *CytB*-*T*-*D*) on the H-strand. The order of these genes in these two clusters was maintained as in the non-rearranged mitogenome of fish, except for the novel location of *tRNA*-*D*. Furthermore, unlike the typical position of the CR in fish, the CR of this species is located between *tRNA*-*D* and *tRNA*-*Q*, thus separating the two gene clusters on the H-strand and L-strand.

**Fig. 1:**
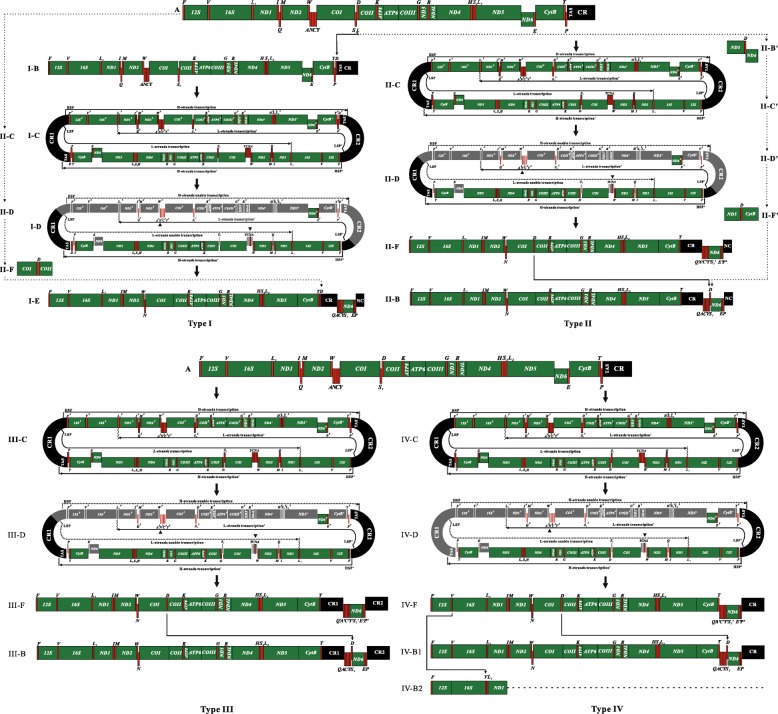
Four mitogenomic rearrangement variants in 13 bothids using the DMNL model. The letters **a** to **f** after the variant number I-IV represent the steps of DMNL process. **a**: Ancestral gene order in typical fish. The rRNA, protein-coding genes and CRs are indicated by boxes; the tRNA genes are indicated by columns, genes labeled above the columns are located on the H-strand, those below the columns on the L-strand. TAS is an acronym for the terminal associated sequences. **b**: Translocated position of *tRNA*-*D* prior to DMNL gene rearrangement in Type I, and that of *tRNA*-*D* posterior to DMNL gene rearrangement and the finally mitogenomic structure in Type II, III, and IV. **c**: The dimeric molecule formed by two monomers linked head-to-tail. The HSP, HSP′, LSP, and LSP′ indicate the H- and L- strand promoters of transcription. The TAS, TAS′, *tRNA*-*L*_*1*_, and *tRNA*-*L*_*1*_′ indicate the H- and L- strand terminations of transcription. The direction of transcription is shown by an arrow. **d**: Functional loss of LSP and HSP. Grey boxes indicate the degenerated genes controlled by non-functional HSP and LSP. The triangle marks the retention of *tRNA*-*N* rather than the expected *tRNA*-*N′* gene. **e**: The translocation of *tRNA*-*D* posterior to DMNL gene rearrangement and the finally mitogenomic structure in Type I. **f**: The mitogenomic structure generated after degeneration of non-transcribed genes in the dimeric molecule. The path from A, II-C, II-D, II-F to I-E in Type I indicates the translocation of *tRNA*-*D* occurred after the DMNL process; the path from A, II-B′, II-C′, II-D′, II-F′ to II-B in Type II indicates the translocation of *tRNA*-*D* occurred before DMNL process; the difference between the steps of B′, C′, D′ and F′ with that of B, C, D and F is the location of *tRNA*-*D*. The broken line in IV-B2 indicates the omitted genes that are the same as those shown in IV-B1

How did this particular mitogenome structure emerge? Four mechanisms have been proposed to account for mitogenomic rearrangements, including duplication-random loss [17], tRNA mis-priming model [18], intramitochondrial recombination [19], and duplication-nonrandom loss [20]. However, none of these four mechanisms can fully explain the gene rearrangements seen in the *C. azureus* mitogenome. Therefore, a novel mechanism called the Dimer-Mitogenome and Non-Random Loss (DMNL) model was proposed to account for the rearrangements found in *C. azureus* [14]. The inferred DMNL process would be as follows, starting with an ancestral mitogenome with a gene order typically seen in fish (Fig. 1a). The first step is a dimerized event of two monomer mitogenomes to form a functionally dimeric molecule linked head-to-tail (Fig. 1i-c). The dual promoter functions in one of two CRs are then lost by mutation and the genes controlled by these promoters are thus no longer transcribed (Fig. 1i-d), and may degenerate. The genes controlled by the remaining two functional promoters continue to be transcribed. Thus, the final gene order in the mitogenome of *C. azureus* is formed (Fig. 1i-e).

**Fig. 2:**
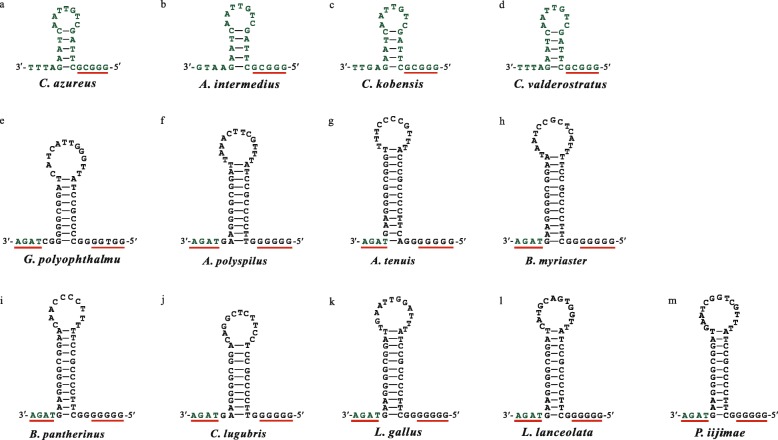
Secondary structures of the O_L_ in 13 bothid mitogenomes. Red underlined bases are conserved sequences. Bases in green font come from the 3′ end of the *tRNA*-*N*. Panel labels (**a**-**m**) represent the corresponding image of O_L_ structure in each of bothids

Retention of *tRNA*-*N* rather than the expected *tRNA*-*N′* gene occurred during the DMNL process. Shi et al. hypothesized that the exceptional retention of *tRNA*-*N* is related to the structure and function of O_L_ based on the study of Seligmann and Krishnan [14, 21]. O_L_ is usually located between *tRNA*-*N* and *tRNA*-*C* of the so-called WANCY region, formed by a cluster tRNA genes that includes *tRNA*-*W*, *tRNA*-*A*, *tRNA*-*N*, *tRNA*-*C* and *tRNA*-*Y* [22]. Due to the rearrangement of *tRNA*-*C* and *tRNA*-*Y*, only a 7-bp intergenic region remained between *tRNA*-*N* and *COI*, an insufficient number of base pairs to allow for the necessary O_L_ secondary structure formation. Interestingly, a 26-bp middle portion of *tRNA*-*N* could form an O_L_-like structure (Fig. 2a) for L-strand replication; this gene was therefore retained [14].

One question remains: when was *tRNA*-*D* translocated from an original site between *tRNA*-*S*_*1*_ and *COII* to a region between *tRNA*-*T* and the CR in the *C. azureus* mitogenome? Shi et al. speculated that translocation of *tRNA*-*D* could have occurred either before (Fig. 1i-b) or after (Fig. 1i-e) the DMNL process [14]. Careful examination and comparison of available mitogenomes of 12 bothid species from eight genera revealed interesting patterns of gene arrangements. Gene organization in these 12 genomes is very similar, except for the shuffling *tRNA*-*V* (Fig. 1IV-B2), and the existence of one of the following four arrangements of CRs, *tRNA*-*D* and the 8-gene cluster (*Q*-*A*-*C*-*Y*-*S*_*1*_-*ND6*-*E*-*P*): *D*–CR–8-gene cluster, CR–5-gene cluster (*Q*-*A*-*C*-*Y*-*S*_*1*_)–*D*–3-gene cluster (*ND6*-*E*-*P*), CR1–5-gene cluster–*D*–3-gene cluster–CR2, and 5-gene cluster–*D*–3-gene cluster–CR.

The DMNL model was also used by Gong et al. [12] to explain the production of the mitogenome of *Bothus myriaster*, the only bothid species other than *C. azureus* for which a mechanism of gene rearrangement has been provided (Fig. 1a, II-c, d, f and b). One difference was noted between the gene structure of the *B. myriaster* and *C. azureus* mitogenomes. In *C. azureus, tRNA*-*D* was translocated outside of the 8-gene cluster (Fig. 1i-e), while in *B. myriaster*, *tRNA*-*D* was found inside this gene cluster (Fig. 1II-B). Whether *tRNA*-*D* translocation occurred before or after the DMNL process was not determined by Gong et al [12].

To better understand the mitochondrial gene structure and gene rearrangement mechanisms of bothids, the mitogenome of the threespot flounder, *Grammatobothus polyophthalmus*, was sequenced and characterized. This species is one of few bothids featuring one lateral line on both sides of the body. We wondered, what are the mitogenomic characteristics of this species? Did rearrangement occur in this mitogenome, and if so, what is the rearrangement type? Our results reveal additional mitochondrial diversity in Bothidae, and provide a foundation for further research on mitochondrial gene rearrangement of fish.

## Results

### Organization and gene rearrangement of the *G. polyophthalmus* mitogenome

**Fig. 3:**
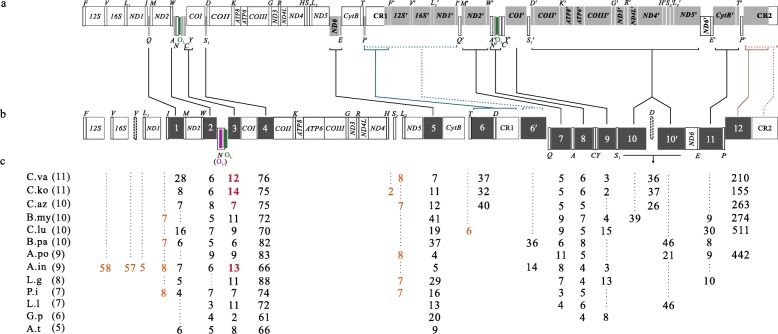
Characteristics of unique intergenic spacers in 13 bothid mitogenomes. **a:** Grey boxes indicate the non-transcribed genes located in the intermediate dimeric molecule. **b:** Dark grey boxes indicate the numbered intergenic spacers. Fourteen loci are numbered as 1 to 12 plus two repeated spacers labeled 6′ and 10′. The lines between images **a** and **b** indicate that the non-transcribed genes in image **a** degenerated to intergenic spacers in image **b**; the pair of blue and brown lines indicate alternative results of degeneration of each underlined non-transcribed genes, respectively. The symbol of  indicates regions of non-transcribed genes. The purple box indicates the O_L_ formed by the middle sequence of *tRNA*-*N*, and the green box indicates the O_L_ formed by an intergenic spacer and 3–5 bp from the 3′ end of *tRNA*-*N*. The *tRNA*-*V* and *tRNA*-*D* in solid and dotted boxes represent the alternative location in four species. **c:** The length of intergenic spacers. Abbreviations of species names are given as follows, P.co: *Pleuronichthys cornutus*; G.p: *Grammatobothus polyophthalmus*; A.t: *Arnoglossus tenuis*; L.g: *Lophonectes gallus*; L.l: *Laeops lanceolate*; P.i: *Psettina iijimae*; C.az: *Crossorhombus azureus*; C.ko: *Crossorhombus kobensis*; C.va: *Crossorhombus valderostratus*; A.po: *Arnoglossus polyspilus*; B.my: *Bothus myriaster*; C.lu: *Chascanopsetta lugubris*; A.in: *Asterorhombus intermedius*; and B.pa: *Bothus pantherinus*. The number in parentheses after the species names is the amount of unique intergenic spacers. Below intergenic spacer No. 3, length of spacers between O_L_ and *COI* are indicated in black numbers, and that between *tRNA-N* and *COI* indicated in dark red. The light brown numbers indicate the length of intergenic spacers that have no relationship to the DMNL process

A total of 18,170 bp of *G. polyophthalmus* mitogenome contained 37 genes, including 13 protein-coding genes, two rRNA genes, and 22 tRNA genes. Among these genes, 28 genes are located on the H-strand, while *ND6* and eight tRNA genes (*N*, *Q*, *A*, *C*, *Y*, *S*_*1*_, *E* and *P*) are located on the L-strand (Fig. 1III-B, Additional file 1: Table S1 and Additional file 2: Figure S1). In this mitogenome, after *tRNA*-*C* and *tRNA*-*Y* were rearranged, a 40-bp intergenic spacer remained between *tRNA*-*N* and *COI*. The secondary structure of O_L_ was formed with 38 bp of this intergenic spacer and 4 bp of the 3′ end of *tRNA*-*N* (Figs. 2e and 3b). The O_L_ had the same 5′-TAGA-3′ sequence motif on the 3′ end as that of eight other bothid species (Fig. 2f–m); the sequence 5′-GGTGG-3′ on the 5′ end was slightly different from either of the motifs 5′-GGGGG-3′ seen in most bothids (Fig. 2f–m), or 5′-GCCGG-3′ seen in most of the 17 flatfishes from seven families [23].

**Fig. 4:**
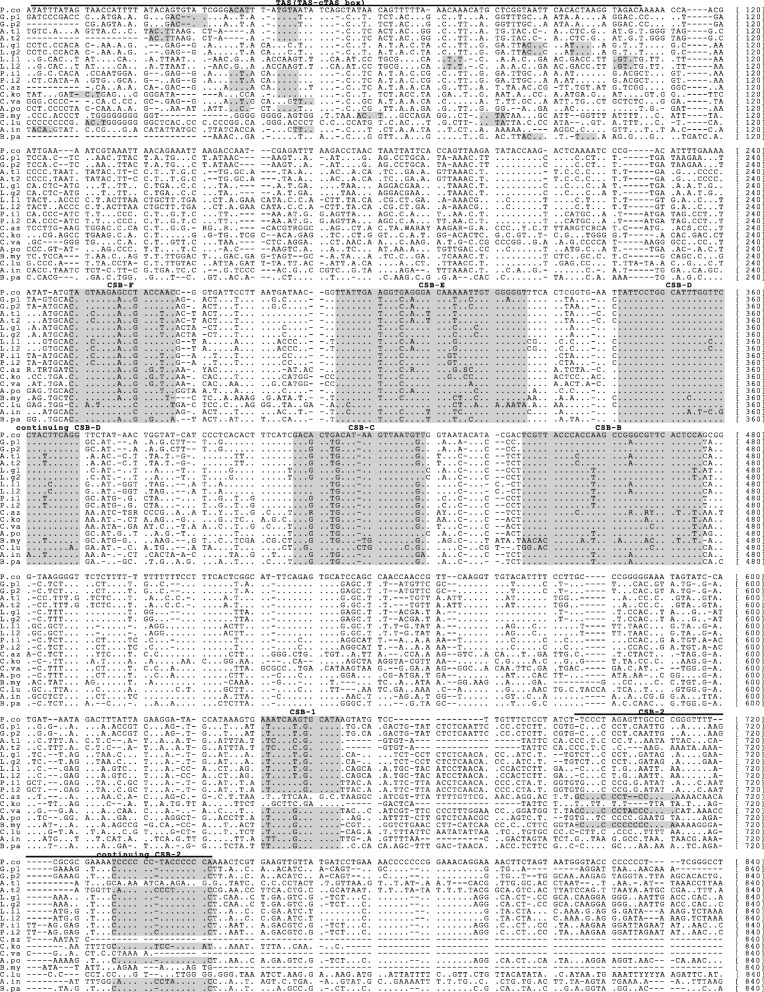
Aligned sequences of control regions in 13 bothids and *Pleuronichthys cornutus*. The shaded blocks represent the conserved sequences. TAS is an acronym for the terminal associated sequence. CSB is an acronym for the conserved sequence block. Species names are abbreviated as in Fig. 3. The number 1 and 2 after the names indicate CR1 and CR2

Two large non-coding (NC) regions were also discovered, NC1 is composed of 773 bp located between *tRNA*-*T* and *tRNA*-*Q*, while NC2 is comprised of 1611 bp between *tRNA*-*P* and *tRNA*-*F*. We compared NC1 and NC2 with conserved CR structures existed in 12 other bothid species and the ridged-eye flounder *Pleuronichthys cornutus*, and found similar conserved structures, including the terminal associated sequences (TAS) with the core sequence ACAT-cTGTA; the conserved sequence blocks (CSB) of central conserved domain, CSB-F, E, D, C and B; the Pyrimidine T-tract; and the other two conserved sequence blocks, CSB-1 and -2 (Fig. 4). Additionally, NC2 had tandem repeated sequences (35 copies of a 22 bp motif) at the 3′ end, as did CR or CR2 in the other species (Additional file 3: Figure S2). Based on sequence conservation of the NCs and the tandem repeats in NC2 of *G. polyophthalmus*, we conclude that both NC1 and NC2 are control regions, thus named CR1 and CR2, respectively (Fig. 1III-B and Fig. 4).

When the previously sequenced mitogenomes of 12 bothid species were compared with that of *G. polyophthalmus*, the gene order of this species was found to be identical to that of four other species, namely, *Arnoglossus tenuis*, *Lophonectes gallus*, *Laeops lanceolata*, and *Psettina iijimae*. This finding raises a question, how were genomic-scale rearrangements and two CRs generated in these five species? Although the mitogenomes of the above four bothids have been reported for years, the mechanism of their gene rearrangement remains unaddressed. Here, using the *G. polyophthalmus* mitogenome as a representative (Fig. 1 Type III), the process of gene rearrangement was found to be consistent with the DMNL model used to explain the evolution of the *C. azureus* and *B. myriaster* mitogenomes.

The process of gene rearrangement in these fishes can be reconstructed as follows, starting with the typical mitogenome of fish (Fig. 1a). First, a dimerized event occurred to form a functionally dimeric mitochondrial DNA (mtDNA) (Fig. 1III-C). At this stage, transcription of genes from the dimeric mtDNA could be initiated normally using the H-strand promoters (HSP and HSP′) and the L-strand promoters (LSP and LSP′) located in the two CRs. Transcription from the H-strand would terminate at TAS and TAS′ in the CRs, while on the L-strand *tRNA*-*L*_1_ and *tRNA*-*L*_1_′ would act as transcription terminators. Second, the function of the promoters (assumed to be LSP and HSP) in one of CRs was lost by mutation, thus the genes controlled by the disabled promoters could not be transcribed and then degenerated or disappeared entirely due to compact feature of mitochondrion (Fig. 1III-D). Consequently, the genes transcribed from LSP′ were clustered together forming the 8-gene cluster “*Q*′-*A*′-*C*′-*Y*′-*S*_*1*_′-*ND6*′-*E*′-*P*′”, and the other group of genes (*F*, *12S*, *V* … *ND5*, *CytB* and *T*) were transcribed from HSP′ (Fig. 1III-F). With the exception of *tRNA*-*N*, genes with identical transcriptional polarity were placed together but were separated by two CRs via the DMNL process. Finally, the gene *tRNA*-*D* was translocated from the site between *COI* and *COII* to between *tRNA*-*S*_*1*_ and *ND6* to form the 9-gene cluster “*Q*-*A*-*C*-*Y*-*S*_*1*_-*D*-*ND6*-*E*-*P*” (Fig. 1III-B).

### Gene-rearrangement mechanism of 13 bothid mitogenomes

In summary, three variants of the DMNL model can be used to account for the gene rearrangements seen in 11 bothid mitogenomes: Type I explains rearrangements seen in *C. azureus*, *Crossorhombus kobensis*, and *Crossorhombus valderostratus*, Type II explains those of *B. myriaster*, *Arnoglossus polyspilus*, and *Chascanopsetta lugubris*, and Type III describes the rearrangement mechanism used in the other five species (Fig. 1 Type I, II and III).

A fourth variant of the DMNL model can also be adopted to explain the rearrangement process that created the *Bothus pantherinus* and *Asterorhombus intermedius* mitogenomes (Fig. 1 Type IV). These two species appear to share the same rearrangement process (Fig. 1a, IV-c, d, and f) except for a difference in *tRNA*-*V* shuffling (Fig. 1IV-B1, B2); in *A. intermedius*, *tRNA*-*V* is moved from the location between *12S* and *16S* (*12S*-*V*-*16S*-*L*_*1*_) to between *16S* and *tRNA*-*L*_*1*_ (*12S*–*16S*-*V*-*L*_*1*_). In Type IV, step A, C, and F of the DMNL processes are similar to those in Type I, II and III; unique to step D of Type IV variant is the degeneration of the whole CR1 (Fig. 1IV-D), leaving only one CR in both the *B. pantherinus* and the *A. intermedius* mitogenomes (Fig. 1IV-B1 and B2).

## Discussion

### *tRNA*-*D* translocation

The *tRNA*-*D* gene is found in one of two sites in the 13 bothid mitogenomes studied here: outside (Fig. 1i-e) or inside (Fig. 1II-B, III-B, IV-B1 and IV-B2) the 8-gene cluster. In the Type I variant, translocation of *tRNA*-*D* to a locus outside of the cluster could occur either before (Fig. 1a, I-B) or after (Fig. 1II-F, I-E) the DMNL process, because either translocation times requires the same number of gene rearrangement steps. In variants Type II, III and IV, translocation of *tRNA*-*D* occurs to the same locus inside the cluster. Using the Type II variant as an example, if the *tRNA*-*D* gene was firstly translocated to a region between *ND5* and *ND6* (Fig. 1II-B′), then after the DMNL process (Fig. 1II-C′, D′) *tRNA*-*D* would be located outside the cluster between *ND5* and *CytB* (Fig. 1II-F′). To become located inside the cluster, this gene would require one more translocation step (Fig. 1II-B). In contrast, if translocation of *tRNA*-*D* occurred after the DMNL process (Fig. 1II-F), one translocation step would be sufficient to create the current gene structure. Therefore, it is more parsimonious to assume that *tRNA*-*D* translocation occurred after, rather than before, the DMNL process in variants Type II-IV.

### The fate of *tRNA*-*N* and *tRNA*-*N′* during DMNL

The *tRNA*-*N* gene is retained in mitogenome of *C. azureus* [14], and Shi et al. hypothesized that this gene acts as a functional O_L_ (Fig. 2a) because the intergenic spacer remaining between *tRNA*-*N* and *COI* is too small to form the necessary O_L_ secondary structure. In the mitogenomes of 12 other bothids, both shorter (12–14 bp) and longer (47–55 bp) intergenic spacers were found. As in *C. azureus* mitogenome, the shorter intergenic spacer in *A. intermedius*, *C. kobensis* and *C. valderostratus* (Fig. 2b-d) could also not form the O_L_ secondary structure directly, but the middle sequence of *tRNA*-*N* could form an O_L_-like structure. The longer intergenic spacer in the other nine species (Fig. 2e–m), when including only 3–5 bp from the 3′ end of *tRNA*-*N*, could also form the O_L_ structure, therefore, *tRNA*-*N* appears to assist in O_L_ function. This finding supports the correlation between the *tRNA*-*N* and the O_L_, which lays a foundation for further studying mitochondrial gene rearrangement and replication.

### Evidence for the DMNL model

Compared with intergenic spacers in non-rearranged mitogenomes of four representative flatfishes, *Psettodes erumei*, *Platichthys stellatus*, *Peltorhamphus novaezeelandiae* and *Pelotretis flavilatus*, six unique intergenic spacers (2–61 bp), either at unique locations or of longer length, were found in the *G. polyophthalmus* mitogenome (Fig. 3). The discovery of such intergenic spacer diversity led us to ask whether such spacers also occurred in the other 12 bothid mitogenomes. We found 121 unique spacers in 12 bothids at fourteen loci (number 1 to 12 plus repeated spacers labeled 6′ and 10′); the length of 115 of these spacers ranged from 2 to 88 bp while the length of another six spacers (at the location numbered 12) ranged from 155 to 511 bp (Fig. 3b). What is the origin of these unique intergenic spacers? And what is the significance of these spacers in rearranged mitogenome? As we traced the DMNL process in each of the 13 mitogenomes, a striking finding was that each non-transcribed gene degenerated to a shorter intergenic spacer (Fig. 3a and b). This finding supports the DMNL model as the mechanism of mitochondrial gene rearrangement in these species, as well as supports the existing of dimeric mitogenome in mitochondrion.

Further analyses showed that the intergenic spacers are evolutionarily diverse. For example, in *C. valderostratus* and *A. tenuis*, the numbers of unique intergenic spacers are 5 and 11, respectively. This result indicates that the corresponding non-transcribed genes are progressively degenerating or have completely disappeared in these 13 bothid mitogenomes. The length of each spacer also varies in different species, for example, spacers numbered 2 and 5 range in length from 3 to 9 bp and 4–41 bp, respectively. This result suggests that each of non-transcribed genes degenerated at different rates in different species.

Seven intergenic regions appear to have no relationship to the DMNL process. Three intergenic spacers of the *12S*–*16S*-*V*-*L*_*1*_ region were found only in *A. intermedius* and were generated by the shuffling of *tRNA*-*V*. The double-A spacer between *tRNA*-*H* and *tRNA*-*S*_*2*_ shared the same base as seen at the 3′ end of *tRNA*-*H*. The other three intergenic regions were also found in the non-rearranged mitogenomes of flatfishes and other teleosts.

### Conclusions

In summary, the newly completed mitogenome of *G. polyophthalmus* and the sequenced mitogenomes of 12 other bothids all possessed genomic-scale rearrangements. These rearrangements could be sorted into four types (Type I, II, III and IV), differing in the particular combination of CR, *tRNA*-*D* and 8-gene cluster and the shuffling of *tRNA*-*V*. The DMNL model can be used to account for all the gene rearrangements in all 13 bothid mitogenomes, except for the translocation of *tRNA*-*D* which appears to have occurred after the DMNL process in 10 of these mitogenomes, and either before or after in three others. During the DMNL process, *tRNA*-*N* was retained rather than the expected *tRNA*-*N′* gene, and *tRNA*-*N* appears to assist in or act as O_L_ function when the O_L_ secondary structure could not be formed directly.

A striking finding was that each of the non-transcribed genes has degenerated to an intergenic spacer during the DMNL process. This result provided significant evidence to support the existence of dynamic dimeric mitogenomes and the DMNL model as the mechanism of gene rearrangement in bothid mitogenomes. These findings highlight a rare phenomenon in teleost fish, which not only promotes the understanding of mitogenome structural diversity, but also sheds light on mechanisms of mitochondrial genome rearrangement and replication.

## Methods

### Sampling, DNA extraction, PCR and sequencing

A single specimen of *G. polyophthalmus* was collected from fishing activity in Fangcheng Port, China, conducted by artisanal bottom-trawling, therefore no specific information is available on depth of capture, substrata, or longitude and latitude of the catch. Fresh specimen was stored in crushed ice immediately after collection, and then frozen at − 20 °C in the lab until further processing. Total genomic DNA was extracted from the epaxial musculature of the specimen using the SQ Tissue DNA Kit (OMEGA) following the manufacturer’s protocol. Nine primer pairs were designed for amplification of the mtDNA genome (Additional file 4: Table S2), based on the known complete mitochondrial sequences of bothids. More than 70 bp of overlaps between sequenced fragments ensured correct assembly of the complete mtDNA genome.

PCR was performed in a 25 μl reaction volume containing approximate 50 ng DNA template, 0.5 μM of each primer, 1.0 U Taq polymerase (Takara, China), 2.0 mM MgCl_2_, 0.4 mM of each dNTP, and 2.5 μl of 10× Taq buffer. The PCR cycling protocol included one cycle at 95 °C for 3 min (initial denaturation), followed by 35 cycles at 95 °C for 30s (denaturation), 45–55 °C for 50 s (annealing) and 68–72 °C for 1.5–5 min (extension), followed by a final extension at 68–72 °C for 10 min. The PCR products were purified by the Takara Agarose Gel DNA Purification Kit (Takara, China) and then were used directly as templates for cycle sequencing reactions. Sequence specific primers were designed for walking across each fragment with an ABI 3730 DNA sequencer (Applied Biosystems, USA). Sequenced fragments were assembled to a complete mitochondrial genome using CodonCode Aligner v3 and BioEdit v7 [24]. For large fragments and walking sequences, manual examinations ensured reliable assembly of the mitogenome. The complete sequence of *G. polyophthalmus* mitogenome was submitted to GenBank under the accession number MK770643.

### Sequence analysis

Identification and annotation of protein coding genes and rRNA genes were performed using NCBI-BLAST (http://blast.ncbi.nlm.nih.gov/Blast.cgi). The tRNA genes and their secondary structures were determined using tRNAscan-SE 1.21 [25], setting the cut-off values to 1 when necessary. The secondary structure of O_L_ was identified by using the mfold web server (http://unafold.rna.albany.edu/?q=mfold). The gene map of *G. polyophthalmus* mitogenome was generated by using CGView [26]. Mitogenomes of eight out of 12 bothid fishes used in this study were determined in our lab, including *A. intermedius*, *A. tenuis*, *B. myriaster*, *C. azureus*, *C. lugubris*, *C. valderostratus*, *L. gallus*, and *P. iijimae*, available under the accession numbers MK256952, KP134337, KJ433563, JQ639068, KJ433561, KJ433566, KJ433567 and KP134336, respectively. Mitogenome sequences of four other bothids were retrieved from GenBank, including *A. polyspilus*, *B. pantherinus*, *C. kobensis*, and *L. lanceolata*, using accession numbers NC_024946, NC_024947, NC_024949, and AP014591, respectively.

## Supplementary information

**Additional file 1: Table S1.** Organization of the *G. polyophthalmus* mitogenome. (DOCX 20 kb)

**Additional file 2: Figure S1.** Gene map of the *G. polyophthalmus* mitogenome.

**Additional file 3: Figure S2.** Aligned sequences of complete control region in 13 bothids and *Pleuronichthys cornutus*.

**Additional file 4: Table S2.** Primers used for fragment amplification of the *G. polyophthalmus* mitogenome.

## Data Availability

The sequence of complete mtDNA of *G. polyophthalmus* in this study was submitted to GenBank under the accession number MK770643. Mitogenomes of other 12 bothids fishes (*A. intermedius*, *A. polyspilus*, *A. tenuis*, *B. myriaster*, *B. pantherinus*, *C. azureus*, *C. kobensis*, *C. lugubris*, *C. valderostratus*, *L. gallus*, *L. lanceolata* and *P. iijimae*) were under the GenBank accession numbers MK256952, NC_024946, KP134337, KJ433563, NC_024947, JQ639068, NC_024949, KJ433561, KJ433566, KJ433567, AP014591 and KP134336, respectively.

## Abbreviations

3-gene cluster: Three genes (*ND6*-*E*-*P*) on L-strand grouped to a cluster
5-gene cluster: Five genes (*Q*-*A*-*C*-*Y*-*S*_*1*_) on L-strand grouped to a cluster
8-gene cluster: Eight genes (*Q*-*A*-*C*-*Y*-*S*_*1*_-*ND6*-*E*-*P*) on L-strand grouped to a cluster
CR: Control region
DMNL: Dimer-Mitogenome and Non-Random Loss mechanism of mitochondrial rearrangement
HSP: H-strand promoters
H-strand: Heavy strand
LSP: L-strand promoters
L-strand: Light strand
Mitogenome: Mitochondrial genome
mtDNA: Mitochondrial DNA
NC: Non-coding region
O_H_: Origin for H-strand replication
O_L_: Origin for L-strand replication
rRNA: Ribosomal ribonucleic acid
TAS: Terminal associated sequences, CSB: Conserved sequence blocks
tRNA: Transfer ribonucleic acid
WANCY: tRNA cluster of *tRNA*-*W*, *tRNA*-*A*, *tRNA*-*N*, *tRNA*-*C* and *tRNA*-*Y*
