# Supplementary material for: Mechanisms of gene rearrangement in 13 bothids based on comparison with a newly completed mitogenome of the threespot flounder, Grammatobothus polyophthalmus (Pleuronectiformes: Bothidae)
Source: BMC Genomics. 2019 Oct 30;20:792. doi: 10.1186/s12864-019-6128-9 (PMC6821024; doi:10.1186/s12864-019-6128-9)
Supplement: Supplementary file 1 — Additional file 1: Table S1. Organization of the G. polyophthalmus mitogenome. (DOCX 20 kb) [file 12864_2019_6128_MOESM1_ESM.docx]

**Additional file 1: Table S1. Organization of the *G*. *polyophthalmus* mitogenome.**

| Feature | Start | End | Length | IS* | Anti-  codon | Start Codon | Stop Codon | Strand |
| --- | --- | --- | --- | --- | --- | --- | --- | --- |
| *tRNA*-*Phe* (*F*) | 1 | 68 | 68 | 0 | GAA |  |  | H |
| *12S* | 69 | 1018 | 950 | 0 |  |  |  | H |
| *tRNA*-*Val* (*V*) | 1019 | 1088 | 70 | 0 | TAC |  |  | H |
| *16S* | 1089 | 2787 | 1699 | 0 |  |  |  | H |
| *tRNA*-*Leu*_1_ (*L*_1_) | 2788 | 2860 | 73 | 4 | TAA |  |  | H |
| *ND1* | 2865 | 3842 | 978 | 2 |  | ATG | TAA | H |
| *tRNA*-*Ile* (*I*) | 3845 | 3915 | 71 | 3 | GAT |  |  | H |
| *tRNA*-*Met* (*M*) | 3919 | 3987 | 69 | 0 | CAT |  |  | H |
| *ND2* | 3988 | 5037 | 1050 | -1 |  | ATG | TAA | H |
| *tRNA*-*Trp* (*W*) | 5037 | 5107 | 71 | 4 | TCA |  |  | H |
| *tRNA*-*Asn* (*N*) | 5112 | 5184 | 73 | -4 | GTT |  |  | L |
| O_L_ | 5181 | 5222 | 42 | 2 |  |  |  | H |
| *COX1* | 5225 | 6778 | 1554 | 61 |  | GTG | TAG | H |
| *COX2* | 6840 | 7542 | 703 | 0 |  | ATG | T | H |
| *tRNA*-*Lys* (*K*) | 7543 | 7614 | 72 | 1 | TTT |  |  | H |
| *ATP8* | 7616 | 7783 | 168 | -10 |  | ATG | TAA | H |
| *ATP6* | 7774 | 8457 | 684 | -1 |  | ATG | TAA | H |
| *COX3* | 8457 | 9242 | 786 | -1 |  | ATG | TAA | H |
| *tRNA*-*Gly* (*G*) | 9242 | 9312 | 71 | 0 | TCC |  |  | H |
| *ND3* | 9313 | 9663 | 351 | -2 |  | ATG | TAG | H |
| *tRNA*-*Arg* (*R*) | 9662 | 9730 | 69 | 0 | TCG |  |  | H |
| *ND4L* | 9731 | 10027 | 297 | -7 |  | ATG | TAA | H |
| *ND4* | 10021 | 11401 | 1381 | 0 |  | ATG | T | H |
| *tRNA*-*His* (*H*) | 11402 | 11471 | 70 | 0 | GTG |  |  | H |
| *tRNA*-*Ser*_2_ (*S*_2_) | 11472 | 11538 | 67 | 4 | GCT |  |  | H |
| *tRNA*-*Leu*_2_ (*L*_2_) | 11543 | 11615 | 73 | 0 | TAG |  |  | H |
| *ND5* | 11616 | 13454 | 1839 | 20 |  | ATG | AGG | H |
| *Cytb* | 13475 | 14615 | 1141 | 0 |  | ATG | T | H |
| *tRNA*-*Thr* (*T*) | 14616 | 14687 | 72 | 0 | TGT |  |  | H |
| CR1 | 14688 | 15460 | 773 | 0 |  |  |  | H |
| *tRNA*-*Gln* (*Q*) | 15461 | 15531 | 71 | 1 | TTG |  |  | L |
| *tRNA*-*Ala* (*A*) | 15533 | 15601 | 69 | 4 | TGC |  |  | L |
| *tRNA*-*Cys* (*C*) | 15606 | 15671 | 66 | 0 | GCA |  |  | L |
| *tRNA*-*Tyr* (*Y*) | 15672 | 15742 | 71 | 8 | GTA |  |  | L |
| *tRNA*-*Ser*_1_ (*S*_1_) | 15751 | 15821 | 71 | 1 | TGA |  |  | L |
| *tRNA*-*Asp* (*D*) | 15823 | 15891 | 69 | 4 | GTC |  |  | H |
| *ND6* | 15896 | 16417 | 522 | 0 |  | ATG | TAG | L |
| *tRNA*-*Glu* (*E*) | 16418 | 16485 | 68 | 3 | TTC |  |  | L |
| *tRNA*-*Pro* (*P*) | 16489 | 16559 | 71 | 0 | TGG |  |  | L |
| CR2 | 16560 | 18170 | 1611 |  |  |  |  | H |

*IS indicates an intergenic spacer that refers to noncoding bases between the feature on the same line and that on the line below. Negative IS numbers indicate overlaps.
