# Supplementary figures and images for: Mechanisms of gene rearrangement in 13 bothids based on comparison with a newly completed mitogenome of the threespot flounder, Grammatobothus polyophthalmus (Pleuronectiformes: Bothidae)
Source: BMC Genomics. 2019 Oct 30;20:792. doi: 10.1186/s12864-019-6128-9 (PMC6821024; doi:10.1186/s12864-019-6128-9)

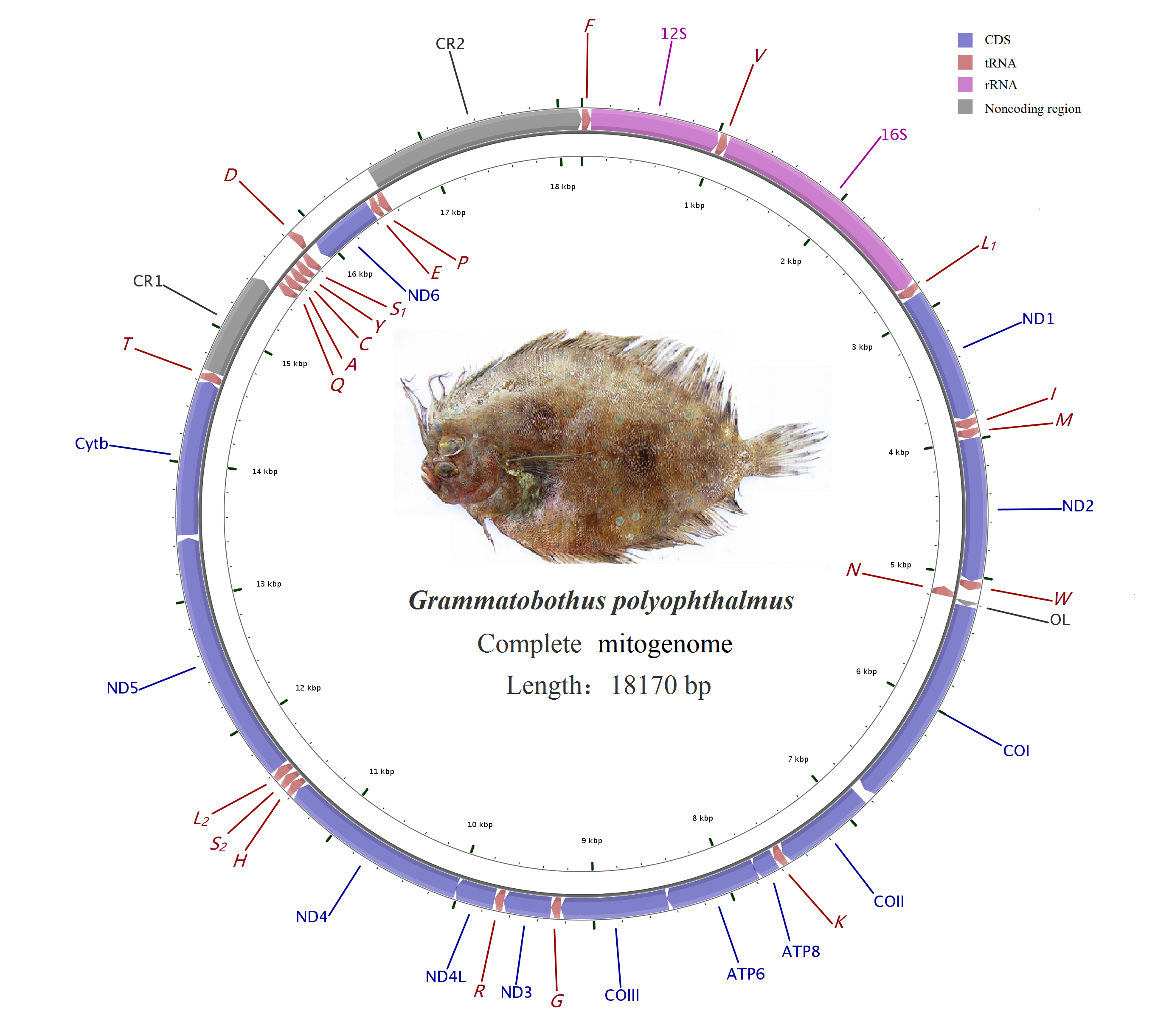

Supplement: Supplementary file 2 — Additional file 2: Figure S1. Gene map of the G. polyophthalmus mitogenome. [file 12864_2019_6128_MOESM2_ESM.png]
