# Supplementary material for: Mechanisms of gene rearrangement in 13 bothids based on comparison with a newly completed mitogenome of the threespot flounder, Grammatobothus polyophthalmus (Pleuronectiformes: Bothidae)
Source: BMC Genomics. 2019 Oct 30;20:792. doi: 10.1186/s12864-019-6128-9 (PMC6821024; doi:10.1186/s12864-019-6128-9)
Supplement: Supplementary file 3 — Additional file 3: Figure S2. Aligned sequences of complete control region in 13 bothids and Pleuronichthys cornutus. [file 12864_2019_6128_MOESM3_ESM.pdf]

```
P.co ---------- ---------- ---------- ---------- ---------- ---------- ---------- ---------- ---------- ---------- ---------- ----------  [ 120]
G.p1 ---------- ---------- ---------- ---------- ---------- ---------- ---------- ---------- ---------- ---------- ---------- ----------  [ 120]
G.p2 ---------- ---------- ---------- ---------- ---------- ---------- ---------- ---------- ---------- ---------- ---------- ----------  [ 120]
A.t1 ---------- ---------- ---------- ---------- ---------- ---------- ---------- ---------- ---------- ---------- ---------- ----------  [ 120]
A.t2 ---------- ---------- ---------- ---------- ---------- ---------- ---------- ---------- ---------- ---------- ---------- ----------  [ 120]
L.g1 ---------- ---------- ---------- ---------- ---------- ---------- ---------- ---------- ---------- ---------- ---------- ----------  [ 120]
L.g2 ---------- ---------- ---------- ---------- ---------- ---------- ---------- ---------- ---------- ---------- ---------- ----------  [ 120]
L.l1 ---------- ---------- ---------- ---------- ---------- ---------- ---------- ---------- ---------- ---------- ---------- ----------  [ 120]
L.l2 ---------- ---------- ---------- ---------- ---------- ---------- ---------- ---------- ---------- ---------- ---------- ----------  [ 120]
P.i1 ---------- ---------- ---------- ---------- ---------- ---------- ---------- ---------- ---------- ---------- ---------- ----------  [ 120]
P.i2 ---------- ---------- ---------- ---------- ---------- ---------- ---------- ---------- ---------- ---------- ---------- ----------  [ 120]
C.az ---------- ---------- ---------- ---------- ---------- ---------- ---------- ---------- ---------- ---------- ---------- ----------  [ 120]
C.ko ---------- ---------- ---------- ---------- ---------- ---------- ---------- ---------- ---------- ---------- ---------- ----------  [ 120]
C.va ---------- ---------- ---------- ---------- ---------- ---------- ---------- ---------- ---------- ---------- ---------- ----------  [ 120]
A.po CGCCAACCCC CTGGGCCTCA ACAGGGCCCT CAGCCGGTGT GGCAGAGCCC GGCTCTCCTT AGGGGCCCTC AGCCGGTGTG GCAGAGCCCG GCTCTCCTTA GGGGCCCTCA GCCGGTGTGG  [ 120]
B.my ---------- ---------- ---------- ---------- ---------- ---------- ---------- ---------- ---------- ---------- ---------- ----------  [ 120]
C.lu ---------- ---------- ---------- ---------- ---------- ---------- ---------- ---------- ---------- ---------- ---------- ----------  [ 120]
A.in ---------- ---------- ---------- ---------- ---------- ---------- ---------- ---------- ---------- ---------- ---------- ----------  [ 120]
B.pa ---------- ---------- ---------- ---------- ---------- ---------- ---------- ---------- ---------- ---------- ---------- ----------  [ 120]

P.co ---------- ---------- ---------- ---------- ---------- ---------- ---------- ---------- ---------- ---------- ---------- ----------  [ 240]
G.p1 ---------- ---------- ---------- ---------- ---------- ---------- ---------- ---------- ---------- ---------- ---------- ----------  [ 240]
G.p2 ---------- ---------- ---------- ---------- ---------- ---------- ---------- ---------- ---------- ---------- ---------- ----------  [ 240]
A.t1 ---------- ---------- ---------- ---------- ---------- ---------- ---------- ---------- ---------- ---------- ---------- ----------  [ 240]
A.t2 ---------- ---------- ---------- ---------- ---------- ---------- ---------- ---------- ---------- ---------- ---------- ----------  [ 240]
L.g1 ---------- ---------- ---------- ---------- ---------- ---------- ---------- ---------- ---------- ---------- ---------- ----------  [ 240]
L.g2 ---------- ---------- ---------- ---------- ---------- ---------- ---------- ---------- ---------- ---------- ---------- ----------  [ 240]
L.l1 ---------- ---------- ---------- ---------- ----ACTCTT GTTGACCGGG CTCTGCCCCG CCAGATGGCA GCAGCCTTTA TGTAAGTGAG CCATCTATGT ATTTACACCA  [ 240]
L.l2 ---------- ---------- ---------- ---------- ---------- ---------- ---------- -CACATGG-- -------TTA TGTAAGTGAG CCATCTATGT ATTTACACCA  [ 240]
P.i1 ---------- ---------- ---------- ---------- ---------- ---------- ---------- ---------- ---------- ---------- ---------- ----------  [ 240]
P.i2 ---------- ---------- ---------- ---------- ---------- ---------- ---------- ---------- ---------- ---------- ---------- ----------  [ 240]
C.az ---------- ---------- ---------- ---------- ---------- ---------- ---------- ---------- ---------- ---------- ---------- ----------  [ 240]
C.ko ---------- ---------- ---------- ---------- ---------- ---------- ---------- ---------- ---------- ---------- ---------- ----------  [ 240]
C.va ---------- ---------- ---------- ---------- ---------- ---------- ---------- ---------- ---------- ---------- ---------- ----------  [ 240]
A.po CAGAGCCCGG CTCTCCTTAG GGACCCTCAA CCGGTGTGGC AGAGCCCGGC TCTCCTTAGG GACCCTCAAC AGGTGTGGCA GAGCCCGGCT CTCCTTAGGG GCTCTCAGCC GGTGTGGCAG  [ 240]
B.my ---------- ---------- ---------- ---------- ---------- ---------- ---------- ---------- ---------- ---------- ---------- ----------  [ 240]
C.lu ---------- ---------- ---------- ---------- ---------- ---------- ---------- ---------- ---------- ---------- ---------- ----------  [ 240]
A.in ---------- ---------- ---------- ---------- ---------- ---------- ---TTAAAGT GTATGCACGC ATGTACATGT ATGCACGCAT GTACATGTAT GCACGCATGT  [ 240]
B.pa ---------- ---------- ---------- ---------- ---------- ---------- ---------- ---------- ---------- ---------- ---------- ----------  [ 240]

                                                                                                                TAS(TAS-cTAS box)
P.co ---------- ---------- ---------- ---------- -------CGC TATAAAATGT TTT-ATAGAC ATATATGTAA TTACACCATA TATTTATAGT AACCATTTTA TACAGTGTAT  [ 360]
G.p1 ---------- ---------- ---------- ---------- ---------- ---------- ---------- -.CAGCCCGG .A.TTGGCAG ATCCCGACCC ..ATGA.A.G ...GAC--..  [ 360]
G.p2 ---------- ---------- ---------- ---------- ---------- ---------- ---------- ---------- ---------- ---------C G.AGTA.A.G ...GAC--..  [ 360]
A.t1 -----TGCCA GTGGCAGAGC CCGGAAGAAC ACGACCCCCC CTGGGGGG.G GTAGGGGG.G GG.ACCCCCA T.TGTA.CGC ...AG.GCAT GTC.A...AG TTA.C..C.T AC.TTAAG.C  [ 360]
A.t2 ---------- ---------- ---------- ---------- ---------- ---------- ---------- ---------- ---------- ---------- ---------- AC.TTAAG.C  [ 360]
L.g1 ---------- ---------- -ACACCTCTC GATTGGCAGG GCCCGGCT-- ----.GGGCC CCAGCCCTGG .CC.TCC--- CCCG.TTT.C CTC.CCACAC -.AA.A.G.. --.GAG--G.  [ 360]
L.g2 ---------- ---------- ---------- ---------- ---------- ---------- ---------- -.T.CCA--- CCCG.TTT.C CTC.CCACAC -.AA.A.G.. --.GAG--G.  [ 360]
L.l1 TTAGTTTATT TTGACCATAC AGGGAATATT GAGGTCTAAG ACGTACC..G ..CG.TG.TC C..G...TTA C..CG..A.. C.CA.GT.C. .G.CAC..TA T....CA... A.TTAAT...  [ 360]
L.l2 TTAGTTTATT TTGACCATAC AGGGAATATT GAGGTCTAAG ACGTACC..G ..CG.TG.TC C..G...TTA C..CG..A.. C.CA.GT.C. .G.CAC..TA T....CA... A.TTAAT...  [ 360]
P.i1 ---------- ---------- ---------- ---------- ---------- AC.TTTGCC. ..GGGGGTGG CAGAGCCCGG .A.AGG..A. .G.C.CACA. CCAATGGA.G --.GAG--G.  [ 360]
P.i2 ---------- ---------- ---------- ---------- ---------- ---------- ---------A CATATCC--- -A.ATG.... CT.CATA-A. GTG..GCA.G --..AG--G.  [ 360]
C.az ---------- ---------- ---------- ---------- ---------- ---------- ------.T.. CAGGC.C.G- -CCACT..A- ---------- .CA..A.A.C A-.GAC--G.  [ 360]
C.ko ---------- ---------- ---------- ---------- ---------- ---------- ---------- --GCCA.G.T CCG.-.A.AT AT...GAT-C .TGAG...GG GGATA-----  [ 360]
C.va ---------- ---------- ---------- ---------- ---------- ---------- --------.. CGG...C.G- -CCA.A...G GG.CCCC--- .CA.C.CC.G C-.GAG--G.  [ 360]
A.po AGCCCGGCTC TCCTTAGGGG CTCTCAGCCG GTGTGGCAGA GCCCGGCTCT CC.T.GGG.C CC.C.ACCGG TGTGGCAG.G CCCGG.T..C CT.CCCCTAC -CAA.A.A.G --..AA-AT.  [ 360]
B.my ---------- ---------- ---------- ---------- ---------- ---------- ---------- ---------- -----TGCG. CCC.ACCCT. TGGGGGGGGG GT--------  [ 360]
C.lu ---------- ---------- ---------- ---------- ---------- ---GGGGCCC CACGCCCCC. CG.C...GGG GGC.T.ACGC CCCCCCCG.A C.TGGGGGGG GCTCAC.CCC  [ 360]
A.in ACATGTATGC ACGCATGTAC ATGTATGCAC GCATGTACAT GTATGCA... ATGT.C.... A.GC.CGC.T G..C.....T GC..G.ATGT ACA.GTAT.C .CG...G.AC ATATTATGC.  [ 360]
B.pa ---------- ---------- ---------- ---------- ---------- ---------- ---------- ---------- ---------- ---------- ---------- ----------  [ 360]

                                 continuing TAS(TAS-cTAS box)
P.co CGGGACATTT -ATGTAATAT CAGCTATAAC AGTTTTTA-A ACAAACATGC TCGGTAATTC ACACTAAGGT AGACAAAAAC CA----ACGA TTGAA---AA TCGTAAATTA ACAGAAATTA  [ 480]
G.p1 ...A..T.A. -.....T... ..-.C..T.T CT.AC...G. C..T...A.G GTTTGC..AA TA.A....AG GAC.CC.... .T-----GAT CCA.C--TC. .AAC.TTACT .A.TG...CT  [ 480]
G.p2 ...A..T.A. -.....T... ..-.C..T.T CT.AC...G. C..TT..A.G GTTTGC..AA TA.A....AG GAC.CC.... .T-----GAT CCA.C--TC. .AA..TTACT .A.TG...CT  [ 480]
A.t1 T-A----.A. -......... ..-.C..TCA CT.AC..GG. C..TT..A.T G.TAC.C-.A ..CTC.G..A T.G.T.GGGT AG----G.CC CCC.TAAT.T ATAC.TT-CC .T..T.G.C.  [ 480]
A.t2 T-A----.A. -......... ..-.C..TCA CT.AC..GG. C..TT..A.T G.TAC.C-.A ..CTC.G..A T.G.T.GGGT AG----G.CC CCC.TAAT.T ATAC.TT-CC .T..T.G.C.  [ 480]
L.g1 A.....T.AC A...C.T... ..-.C..T.A .T.A.C.TA. C..TT..G.G A.TTAC..C. .AT.....AG .T..T....G .C----.AAC A.CTC-ATG. ..T..TT..C .TGA..C.C.  [ 480]
L.g2 A.....T.AC A...C.T... ..-.C..T.A .T.A.C.TA. C..TT..G.G A.TTAC..C. .AT.....AG .T..T....G .C----.AAC A.CTC-ATG. ..T..TT..C .TGA..C.C.  [ 480]
L.l1 -AAC.G..A. ACCAAGT.T. .CA.AT.CCT GCG....CA. .T.T.-.C.. A.A.C..GAA C.GACC.TT. GT.TG.TT.. TT-----AAT ACT..ACCCC .T.ACTTAAC TGCTT.TGA.  [ 480]
L.l2 -AAC.G..A. ACCAAGT.T. .CA.AT.CCT GCG....CA. .T.T.-.C.. A.A.C..GAA C.GACC.TT. GT.TG.TT.. TT-----AAT ACT..ACCCC .T.ACTTAAC TGCTT.TGA.  [ 480]
P.i1 A.....T.AC A......... ..-.C..TCA .T.A.C.CG. C..TT..G.G A.TTGC..AA .AT......G .ACGCT...G T.----.AAC A.CCC-ATCT .T...CCC.C .T..G.T.C.  [ 480]
P.i2 A.....T.AC A......... ..-.C..TCA .T.A.C.CG. C..TT..G.G A.TTGC..AA .AT......G .ACGCT...G T.----.AAC A.CCC-ATCT .T...CCC.C .T..G.T.C.  [ 480]
C.az ...A....AC A.......T. ..-.C..TCA .T.A.A.CA. G..TT..A.T .TA.AC.CG- TT.TGT.ATG .TCGG....G GC----..AT CCTTG-AAGT GGAC.C.CAC .T..G.G.-R  [ 480]
C.ko --A...CCA. -......... ..-.C..T.T CT.ACC.TA. GA..T..G.A AT.A..CC.A .ATGA.G..A T....GT.GG ..----TTC. ..CG.AGCCT GAAG.C.A.C .T......G.  [ 480]
C.va ...A..T.GC A...GTT... ..-.C..GCA .T.A.A.CA. C......A.T .TT.GC..AA TT.TG..CTG CTCTC...GG A.----.TA. GC----GGGT G....C.A.C .T..GTT.C.  [ 480]
A.po ATT...C.-C T.....T... ..-.C..CG. TT.A.A.GA. G..TT..G.G G.--A..GA. .AC....... ....T.G.-G AT----.AAC CC.GT-AT-. ..AG.CCC.T .T....T.C.  [ 480]
B.my ----.GGGGG GGG.AGTGG. T.TAA.AC.T ...GCCAGAG G..CT-...T ATAA...GCA TT--GGTTTA TTTT...C.A TT----TA.T C.TCCA---T .AA.C..AGT .T.TTGGACT  [ 480]
C.lu .CCCGG.CA. GGG.ACCCTC .C..CCATGT .C.AC...T. G.CTT-...T ATTA.C.CCA TA--..GT.A .TTT...C.- T.----CA.G .CCC.A---T .TA.CCTA-C .T.TTGTAC.  [ 480]
A.in TTATCACCA. -...CTT... ..-.C...T. CT.A.A.TA. .T.CT..A.A AAAA..---A GGC.C....G TT...T..TA .C----C.AC ACC.TAATCT CT-C.TT-CG .TGA.TC.C.  [ 480]
B.pa -AAAC..GA. -.....T..A ..-.C...GG TT..A...G. ....T..G.A C.TTAC.... T.-T.....AA G.G...G... .TGATC.A.C .CACG---.. GA.C.TGG.. G...T--C..  [ 480]
```

```
                                                                                                                                        CSB-F
P.co AGACCAAT-- -CGAGATTTA AGACCTAACT AATTATTCAC CAGTTAAGAT ATACCAAG-A CTCAAAATCC CG-----ACA TTTTGAAAAA TAT-ATGTAG TAAGAGCCTA CCAACC--GG  [ 600]
G.p1 .ATAAC---- -AAGT..A.- .AT....G.A G.CCTGCA.T A-AAAC.TT. ........C. ...C.CT..T .-----TGAT AAGAA...TT A-GTGCAC.. ....A..G.. .....AG-.A  [ 600]
G.p2 .ATAAC---- -AAGT..A.- .AT....G.A G.CCTGCA.T A-AAAC.TT. ........C. ...C.CT..T .-----TGAT AAGAA...TT A-ATGCAC.. ....A..G.. .....AG-.A  [ 600]
A.t1 --TG.GC.A- --.TA.A... .AC..A.CA. TC..GA.A.G TTAAAC.T.. ........-T .C.C.C...T .T-----GA. G...CCCC.. ..ATGCAC.. C...A..G.. ..T..AC-TA  [ 600]
A.t2 --TG.GC.A- --.TA.A... .AC..A.CA. TC..GA.A.G TTAAAC.T.. ........-T .C.C.C...T .T-----GA. G...CCCC.. ..ATGCAC.. C...A..G.. ..T..AC-TA  [ 600]
L.g1 ..TA..---- -.A.T.GA.. ...TAA...A GGACGAA... .TAAAC.T.. ........C. .C...C..AT .-----TGA. A.A.T.G... A.ATGCAC.. ....A..G.. ..T..ACTA-  [ 600]
L.g2 ..TA..---- -.A.T.GA.. ...TAA...A GGACGAA... .TAAAC.T.. ........C. .C...C..AT .-----TGA. A.A.T.G... A.ATGCAC.. ....A..G.. ..T..ACTA-  [ 600]
L.l1 .CT.A.GAAC ATA.C.C.A- CTT.TA.CA. CG.CT.GA.. A-AAACG... .C......T. ...C.T...T .T-----GTG .A..C...T. ..GTGCAC.. C...A..G.. .....G--TA  [ 600]
L.l2 .CT.A.GAAC ATA.C.C.A- CTT.TA.CA. CG.CT.GA.. A-AAACG... .C......T. ...C.T...T .T-----GTG .A..C...T. ..GTGCAC.. C...A..G.. .....G--TA  [ 600]
P.i1 ..TT..---- -AA.AT.G.G .AGTTA...A GC.C.A...T ..AA.C.T.. ........T. .CATGC..A. T----ATGAT AG.CCT..TT A-ATGCAC.. C...A..G.. ..T..G--A.  [ 600]
P.i2 ..TT..---- -AA.AT.G.G .AGTTA...A GC.C.A...T T.AA.C.T.. ........T. .CATGC..A. T----ATGAT AG.CCT..TT A-ATGCAC.. C...A..G.. ..T..G--A.  [ 600]
C.az G..R..---- CACGTRGGC. .AG..CT.AC .TA.WARAYA AGAR-G.A.C CC..Y.C.TT TARGTCRCAT .C---ATG.. ACS.CC..TR .TRTGATC.. ....A..G.. G.T..AA-.Y  [ 600]
C.ko G-TG..TCGC --ACA.GAG. .TC..CT.GG T..C.A.T.G G-ACACTC.G .C.GT..T-C .G....TGGGG .T-----.AC AC.GAC.CT. CC.TGA.C.. ....A..G.. G.T..AA-.C  [ 600]
C.va ..TAA.---- CTC.AGGA.. .CT.AAC.AA ...C.AAG.A .T.G-C.CGC CCGG..G.A. A.G.CCCCAT -----AAGGG CC..CC..T. AT.TGA.C.. ....A..G.. G.T..AA-.C  [ 600]
A.po ..-A.C---- -AAGTC.A.. .A..TC...A T.AA.A...T GTTGAC.CC. .C......T. .ATG.C..AT .-----TGA. AC-.A.C.TG AG.TGCAC.. ....A..G.. ..T..GGTAA  [ 600]
B.my ...GAC.GAG -TAGT--GC- .A.TA...A. TC.C..GA.. TCAAAC.T.. ........TC TC..GC.C.T .C------GC AGC.C...G. AG.TGCTC.. ....A..G.. ...T---CT  [ 600]
C.lu .ATTA.GATT -TA.TT.AC- .ATT.A.CA. ...A..CT.. TTAACC.T.. ........T. GAG.CC...T .TTA-TA.AC ...G...C.TG AG.TGG-C.A .T.....C.. .....TGA-CT  [ 600]
A.in --.G.TCCC- --AC.G..CC GTC..G.TA. ...AGA--.T TTAAAC.T.. .C.A...G.-. .....C.-.A .T-----TGG A..CA.GC.. ...ATGCAC.. .........C.. .....AG-.A  [ 600]
B.pa GT..AC.A-- -..CT..... .T...-.... C.AAG.C.G. G.A--.CAC. C.......-T A....C.A.A AA-----TG. A.G.C....G G..TGCAC.. ....A..G.. G...A.--TA  [ 600]
                                            CSB-E                                                          CSB-D
P.co TGATTCCTTA ATGATAAC-- GGTTATTGAA GGTGAGGGAC AAAAATTGTG GGGGTTTCAC TCGGTG-AAT TATTCCTGGC ATTTGGTTCC TACTTCAGGT TCTAT-AACT GGTAT-CATC  [ 720]
G.p1 CT...T.... .G.C....-- .........T ...C.A.... ..T..C.... .A........ .TA...-..C .......... .......... .........G C.AT.--.A. A.G.CTT--T  [ 720]
G.p2 CT...T.... .G.C....-- .........T ...C.A.... ..T..C.... .......... .TAA..-..C .......... .......... .........G C.AT.--.A. A.G.CTT--T  [ 720]
A.t1 CT...T.... ........-- .........T ...C.A..T. ..T....... .......... .TA...-C.C .......... .......... ....T..... A.AC.-C.T. TA.T.-G-..  [ 720]
A.t2 CT...T.... ........-- .........T ...C.A..T. ..T....... .......... .TA...-C.C .......... .......... ....T..... A.AC.-C.T. TA.T.-G-..  [ 720]
L.g1 CT...T.... ...C....-- .........T ...C...... ..C...C... .......... ..A...-... .......... .......... .......... C.AT.-.T. .A...-T..T  [ 720]
L.g2 CT...T.... ...C....-- .........T ...C...... ..C...C... .......... ..A...-... .......... .......... .......... C.AT.-.T. .A...-T..T  [ 720]
L.l1 .....A.... ..ACCC..-- .T.......T ...C.A.... ..G....... ....CG.... C.C...-..C .......... ....C..... ...C.....G ..AT.-GGT. TAG..---.AT  [ 720]
L.l2 .....A.... ..ACCC..-- .T.......T ...C.A.... ..G....... ....CG.... C.C...-..C .......... ....C..... ...C.....G ..AT.-GGT. TAG..---.AT  [ 720]
P.i1 .T...T.... ...CC...-- .........T ...C...... .GT....... .......... CTA...-..C .......... .......... ....T....G C.ATG-.G.C TA....-...T  [ 720]
P.i2 .T...T.... ...CC...-- .........T ...C...... .GT....... .......... CTA...-..C .......... .......... ....T....G C.ATG-.G.C TA....-...T  [ 720]
C.az AC........ ...-AT.AC- .........T ...C.R.... ..G.SC.... .........A .TCTA.-C.. .......... .......... .........A A.ATC-TSRC CCG..A..AT  [ 720]
C.ko AC...T.... ...CATGG-- CC.......T ...C...... .G..CC.... .........A CTCC..AC.. .......... .......... .........A A.AT.-.CTA .AG..-G...  [ 720]
C.va AC...AA.... .G.CATGG-- CC.......T ...C...... ......A... .........A .ACT.A-C.. .......... .......... .........A A.ATA-.GAA T.C..-T.AA  [ 720]
A.po .T...A.... ...C....-- .........T ...C.A.... ......C... ....A..... C.C...-... .......... .......... .........G C.AT.G..T. .A.G.-T...  [ 720]
B.my C..A.AAAG. G.ATA.T.-- .T.......T ...C...... ..CT..C... ....AA.... A.A...-..C .......... ..C....... ....T.....G C.ATG-G..T. .AAG.--.GT  [ 720]
C.lu CC.C...AGG G..TA..GA- .A..C...... .......... ..CT..A..A .AATA.A..A A.....-..C .......... .......... ........A.G A.AT.-.C.A .TG..--.AT  [ 720]
A.in CT...T.... ..AC..T.-- .T..C....T ...C....T. ..TC...... ....C..... A.....-... .......... ..A.T-C.GA .T........A A.A-.-.CT. CACTA-A-C.  [ 720]
B.pa CT..AT...T ...CA...-- .T.......T ...C....T. ..T..C.... ....C..... A.TA..-... .......... .......... .........G A.-..-GC.. T.G..A.G..  [ 720]
                           CSB-C                                                 CSB-B
P.co CCTCACACTT TCATCGACAC TGACAT-AAG TTAATGTTGG TAATACATA- CGACTCGTTA CCCACCAAGC CGGGCGTTCA CTCCAGCGGG -TAAGGGGTT CTCTTTT-TT TTTTTTCCTT  [ 840]
G.p1 ..G....... .........G. .TG...-... ........G.. A..A-...C- -CC....... .......... ..A....... .....TA... -C.TCT.... .CT....T.G ..C.--....  [ 840]
G.p2 ..G....... .........G. .TG...-... ........G.. A..A-...C- -CC....... .......... ..A....... .....TA... -C.TCT.... .CT....T.G ..C.--....  [ 840]
A.t1 .....TT... .........G. .TG...-... ........G..T ...C-...C- -TCT...... ...C...... ..A....... .....CA... -CC.TTT.G. TCTC...T.. A..C.C.-..  [ 840]
A.t2 .....TT... .........G. .TG...-... ........G..T ...C-...C- -TCT...... ...C...... ..A....... .....CA... -CC.TTT.G. TCTC...T.. A..C.C.-..  [ 840]
L.g1 ....G..... ....T...G. ..G...-... .........C. A..C-...C- -CCT...... ......T... .......... ....T.AT... -C.TTT.... .......T.G G...--....  [ 840]
L.g2 ....G..... ....T...G. ..G...-... .........C. A..C-...C- -CCT...... ......T... .......... ....T.AT... -C.TTT.... .......T.G G...--....  [ 840]
L.l1 ..A....... .......... .TG...-... ........G.. ...C-...-C .-CT...... ......T... ..A....... .....CA... GC.GCT.... .-.....-.. AGG.......  [ 840]
L.l2 ..A....... .......... .TG...-... ........G.. ...C-...-C .-CT...... ......T... ..A....... .....CA... GC.GCT.... .-.....-.. AGG.......  [ 840]
P.i1 ..G..T..G. .........G. .TG...-... ........G.. C..C-...C- -CCT...... ......T... .......... .....T.... -C.TCT.... .CT....TC. C...-.....  [ 840]
P.i2 ..G..T..G. .........G. .TG...-... ........G.. C..C-...C- -CCT...... ......T... .......... .....T.... -C.TCT.... .CT....TC. C...-.....  [ 840]
C.az ..Y..T..G. C...T...R. .TG...-... ........G.A Y..C-...C- -TCT...... ...C...... ..R..RY... T..T.AA.TA -C.TCT.... .YTC...T.C .C........  [ 840]
C.ko ..G..TC..A .G.C....G. .TG...-... ........G.A A..C-...C- -ACT...... .......... ....A..... ....T.AA... -C-.TT.... A.T....A.. A......AA.C  [ 840]
C.va ..C..T..G. C...T...G. .TG...-... ........G..T ...C-...C- -ACT...... .......... .......C.. ....T.AA... -C.TTT.... .CT....T.C .C..A.....  [ 840]
A.po ....T..... ....T...G. .TG...-... .........C. A..C-...C- -ACT...... ......T... .......... ....T.AG... -C.TTT.... .CT....T.. .C..A.....  [ 840]
B.my ..C..TCGA. CG.CT...G. .TG...-TG. ........G.. AG.C-...TA TA.TAACAC. ....A.T... A....AC... T..T.-A... AC.TAT...A G......T.. A.........  [ 840]
C.lu A....T.... ...CT..G.. ......CTG. .......C.G.. ...C-..CGT --.TGG.AC. .......... ......C... .....CA... GC..TTA... .C.....A.. ......C.T..  [ 840]
A.in ....TA..G. .T........G. .TG...-... ........GA. A..C-...C- -TCT...... ......T... ..A....... .....CA... GCCTCT.... .CT....TC. ..........  [ 840]
B.pa ..A..T.... C...T...G. .TG...-... ........G.. A..C-...-- .TCT...... ......T... ..A....... .....CA.C. -C.TCT...A --C....-.. .A..A.....  [ 840]

P.co TCACTCGGCA T-TTCAGAGT GCATCCAGCC AACCAACCGT T--CAAGGTT GTACATTTTC CTTGC----C CGGGGGAAAT AGTATC-CAT GAT--AATAG ACTTTATTAG AAGGA-TA-C  [ 960]
G.p1 .....T.A.. .-........ ...GAGC.T. T--ATGTTCG C--....... ..T.....C. T-..----T ...CAC.GTA .TG-.G-A.. .G.G-..A.. ..A.ACCGTC ...AG-.T-G  [ 960]
G.p2 .....T.A.. .-........ ...GAGC.T. T--ATGTTCG C--....... ..T.....C. T-..----T ...CAC.GTA .TG-.G-A.. .G.G-..A.. ..A.ACCGTC ...AG-.T-G  [ 960]
A.t1 ...A.T.... .-........ ...G.GC.T. T.---TGTTA .ATT...... .AT......T GG..-----. .A..CC.C.. GTA..GTA.. .C.TTT.A.C .T.C--.A-T ..A..A.T-G  [ 960]
A.t2 ...A.T.... .-........ ...G.GC.T. T.---TGTTA .ATT...... .AT......T GG..-----. .A..CC.C.. GTA..GTA.. .C.TTT.A.C .T.C--.A-T ..A..A.T-G  [ 960]
L.g1 .....TTA.. .-........ ...GAGC.T. T--ACGA.TA .--....... .......... T-..-----. T.CCAC..TA ...G..-AT. TC.--T.AG. .TAA.C...T ...A..-.T-G  [ 960]
L.g2 .....TTA.. .-........ ...GAGC.T. T--ACGA.TA .--....... .......... T-..-----. T.CCAC..TA ...G..-AT. TC.--T.AG. .TAA.C...T ...A..-.T-G  [ 960]
L.l1 ...ACTT... .-........ ...G.GC.T. T.T-G.TATA .--....... .AG...C... ....T----T T.TAAC...G .TA..G-A.. .C.--.GAG. ..A.GTC..T ...A.-CC-A  [ 960]
L.l2 ...ACTT... .-........ ...G.GC.T. T.T-G.TATA .--....... .AG...C... ....T----T T.TAAC...G .TA..G-A.. .C.--.GAG. ..A.GTC..T ...A.-CC-A  [ 960]
P.i1 .....T.... .-....C... ...AGGCATT .--A..A.AA A--T...... ..C.....C. .C..-----. ..CCAC..GA .TGT.A-ACG CT.--.GAG. .TA..C.GCT ...A.-.T-G  [ 960]
P.i2 .....T.... .-....C... ...AGGCATT .--A..A.AA A--T...... ..C.....C. .C..-----. ..CCAC..GA .TGT.A-ACG CT.--.GAG. .TA..C.GCT ...A.-.T-G  [ 960]
C.az ....CTT... .GGG.CTG.. TGT..A.TTA .--.CCA.AA G--GTC..AC A...T..GAT TG.C.----. T.CTACT... -TAG.G-A.. .T.--..A.. ..AA.C.A-C ...AG-C.-G  [ 960]
C.ko ...GG.AA.. .---..CA--. ....AGCTAA GGTA-CGTTA A--......C .G....A.C. .G..G----T .TA.CC.C.. .AAG.G-A.. .--TT..AG. ...C--.A.T ..A.TG.TTA  [ 960]
C.va .....TTA.G CGCC..TGA. CATAAGTAAG .--G.GA.AA G--GGC..AC AA.TTC.GA. TGAC.----G A.C.AT.--- -TGG.G-A.. .G.--G.A.. ..A..CAA.C ...A.-..-A  [ 960]
A.po ...CTT.... .-C...C... ...GGA.... CG-ATGA.TG A--....... .AT...CACT T-..-----A TA.AA-..CG G...G-A.. .TC--.GG.A ..A..GA..C ...AGGCT-G  [ 960]
B.my ...-CAAA.. .-G.....-. .T.---...T C..A..ATAA .--GG.AAG. .A....GA.- --------G. .CC.....C.C .TGT.G-A.. ..AT-..A.. ..A.AGCCG- G.CA.-CC-A  [ 960]
C.lu ...GCTT... .-....C... .TT---.AAT C.GA..AAA. C--A...... .G.....AACT G.C.TACCA. TCA..T.... GTGG.G-A.. ...G-T.A.. T.A....C.GT ....G-CT-G  [ 960]
A.in ...A.TTA.. .-C....... .....AAC.T. ..-AG.A.TG G--T...... .GT..C..A. .....----A .A..CG.GG .A....TTA. ..ATG...T. .T.CAT.A.. .TCA.G.TTG  [ 960]
B.pa ..CTCTTA.. .-........ ...GGGC.T. ..--.G.TAG C--....... ...T...G.C. .....----A .C.CAAC..G .TGG.G-A.. ....T-..A.. ..A..GA-.T ...A.--G-G  [ 960]
```

```
                          CSB-1                                                                                 CSB-2
P.co CATAAAGTGA AATCAAGTGC ATAAGTATGT CC-------- --------TT GTTTCTCCTA TCT-TCCCTA GAGTTGCCCC GGGTTTT--- ----CGCGCG AAAATCCCCC C-TACCCCCC [1080]
G.p1 ...T...AT. T....T.G.. ....TG.CA. GACTG-TATC TCTCAATTC. CC.CTCTT.C GTG-C...CC CT.CAATTG. .A....AAG- ----GAAAG. T...C..... .-........ [1080]
G.p2 ...T...AT. T....T.G.. ....TG.CA. GACTG-TATC TCTCAATTC. CC.CTCTT.C GTG-C...CC CT.CAATTG. .A....AAG- ----GAAAG. T...C..... .-........ [1080]
A.t1 ..ATTTA.T. TC...C.G.. ....------ GTGT-A---- ---------- ----TATTCC A.CCC.T.CC C.T..AATAT TAC...CA-- --------AT ...GCA.AAA TCA.AGA..G [1080]
A.t2 ..ATTTA.T. TC...C.G.. ....------ GTGT-A---- ---------- ----TATTCC A.CCC.T.C. C.---AAG.A AATA.AAA-- --------AT GGTT.A.... .CCCT..... [1080]
L.g1 ..AT...A.. T....T.G.. ....CA-... ..TCT-CCTC TCTCAACA.C C..ATC.T.- -TGTCT..CC CT..GATAG. A....GAA-- ----AAA... T...C..... .-........ [1080]
L.g2 ..AT...A.. T....T.G.. ....CA-... ..TCT-CCTC TCTCAACA.C C..ATC.T.- -TGTCT..CC CT..GATAG. A....GAA-- ----AAA... T...C..... .-........ [1080]
L.l1 ..CT..AG.. T....T.G.. ....CAGCA. A.TGC-TACA TCCTAACA.. CCACTCTT.G A..-C...CT .G..AATT.A A......A-- ----ATG.G. T...C..... .-........ [1080]
L.l2 ..CT..AG.. T....T.G.. ....CAGCA. A.TGC-TACA TCCTAACA.. CCACTCTT.G A..-C...CT .G..AATT.A A......A-- ----ATG.G. T...C..... .-........ [1080]
P.i1 ..AT..T.A. T.....G.. ...TTAC.A. A.TTC-TTAA CCTCAACA.C CC.CTA.T.G GTGTG...CC CG.G.ATAT. A...C.AATT T--AG.GAG. T...C..... .-........ [1080]
P.i2 ..AT..T.A. T.....G.. ...TTAC.A. A.TTC-TTAA CCTCAACA.C CC.CTA.T.G GTGTG...CC CG.G.ATAT. A...C.AATT T--AG.GAG. T...C..... .C........ [1080]
C.az -.C.T.TAA. T..TTCAA.G .C.TAAGGC. ATCGT-TTAT TTTGTTCG.A AC.AGG.ACT .T.GC...CC CT--CC.... AAAACAACAT ----AATATC ---------- ---------- [1080]
C.ko .....TTCT. .GCTTC.GA. ....------ GACTCA---- ---------- ----TATTCT ...T..TT.T .T----TTAT A.T...AG-- -------.AAT TTTGC..... .TCC-....A [1080]
C.va ..C...T... T....G.... ...TAC..C. ATCGT-TTCC CCTTTGGAAC C..GGATGGT .TACC...CC CTACCC.... CAT.AAACCC ----G..A.C T.C.CTAAAA .--------- [1080]
A.po ..ACCTT.A. T....G.... .....AG... ATTTT-CTTG TCTCAACGC. ..AGTC.T.- -TGTG..TCC CCC.GAATGT A....AGA-- ----AAAAG. T...C..... .-........ [1080]
B.my ...T..ACT. T....G.... ...-CGT... GGTCTGAACC TT-CATCAAC C.C..CTTCG GTA-C...CC CCCCCC...A AAAAGGGA-- --ATA---TA TT...AGAA- --A....AGT [1080]
C.lu ...T..TCT. T....G.... ...-CAG.A. T.TTTATTCA ATATATTAA. TC..TGTGCC C.C-CTT.C. CCC..TTTTA A.....AG-- ---C.C---C CT.CC...GT --...TTGGG [1080]
A.in .....TT.A. TT...G.... ...TTG-.A. GTTTTAA--- --------C. .GACTAGTAT CT.G..TAAG .GCC.TAA.T AACG.AAA-- -------ATT TTGG.A.... .CCTA..... [1080]
B.pa ...TCTA.TT T....G.... .....C.CAC A.AGC-TTTG AC-TAACA.. ACC.TCTTCG ..C-C....G C...AA.AAA ..TC.AGA-- ----.TA.G. T...C..... .-........ [1080]

P.co AAAACTCGTG AAGTTGTTAT GATCCTGAAA ACCCCCCCGG AAACAGGAAA ACTTCTAGTA ATGGGTACCC CCCCCT---- -TCGGGCCTT TTGGCCCTAA TATTTA-TAA TACAA-CAAA [1200]
G.p1 CTT.A..C.A ..ACATC..A -CAGT--..G C....--... ........G. TG..A----- ----AGGATT AA..AACAAA ---------- ---------- ---------- ---------- [1200]
G.p2 CTT.A..C.A ..ACATC..A -CAGT--... C....--... ........G. TG..T--TGG CAA.AAGAGT AGGTA.TTAA GCACACTG.A A.TAAAGA.C .G.GCTTA.. C.AT.GTTC. [1200]
A.t1 .G..TATC.C .C.CCTACT. T.GTTAA.GT ..T.AT-.AA .T---CACG. TTG.GC.ACC TAAT--..T. AA.-.AT--T AAACCTTAA- ---------- ---------- ---------- [1200]
A.t2 CCG.AA.CCT TCA.CTG.C. .GCATAAT.T .T..TA-TAT .T.T.TACGG CA.GTC.ACT TATCCAG.TT AA(ACATGCC GACGTTTCAG AGGCGTTTTT ATTGTACGAC TGCTCATTGT [1200]
L.g1 CT.CAA..CT .GA.GTC.G. -TCT...C.. ....--.... ......AGG. CA.CT-.CCG CA.CAAGGAG GG..AATTGA CC.CAC..AA ---------- ---------- ---------- [1200]
L.g2 CT.CAA..CT .GA.GTC.G. -TCT...C.. ....--.... ......AGG. CA.CT-.CCG CA.CAAGGAG GG..AATTGA CC.CAC..CA GGCTTGTAC. A(GCACTATA TAGTGGTGCC [1200]
L.l1 CT..A..C.C .GA.ATC.T. .......C.. .....--... ........G. TA.CTA...C .AAA.G.AGG .GATA---AA AG.TCTAAAA GCCATGTC.. CGACAGGG.. ACA.GAAG.G [1200]
L.l2 CT..A..C.C .GA.ATC.T. .......C.. .....--... ........G. TA.CTA...C .AAA.G.AGG .GATA---AA AG.TCTAAAA GCCATGTC.. CGACAGGG.. ACA.GAAG.G [1200]
P.i1 CT..AAT..A .GACGTC.G. -TCT...C.. ....--.... ......AGG. C..CT..CC. TA..AAGAAG GATTAGAATA T..AAC..CA CCCCA..C.. ---------- ---------- [1200]
P.i2 CT..AAT..A .GACGTC.G. -TCT...C.. ....--.... ......AGG. C..CT..CC. TA..AAGAAG GATTAGAATA T..AAC..CA CCCCA..GGC ..C...T... GTAGCTGT.C [1200]
C.az ---------- ---------- ---------- ---------- ---------- ---------- ---------- ---------- ---------- ---------- ---------- ---------- [1200]
C.ko T...AAAT.T TTA..CAA.- ..C.------ ---------- ---------- ---------- ---------- ---------- ---------- ---------- ---------- ---------- [1200]
C.va ---------- ---------- ---------- ---------- ---------- ---------- ---------- ---------- ---------- ---------- ---------- ---------- [1200]
A.po CT..AA..CA .GA.GTC.G. -TCT...C.. ....--.... ......AGG. CA.CT..CA. TA...AGGAA GGT.AAC--- CA.AAC...C AATT.----- ---------- ---------- [1200]
B.my G--------- ---------- ---------- ---------- ---------- ---------- ---------- ---------- ---------- ---------- ---------- ---------- [1200]
C.lu G.GGG.TAAA TCT.AAG.GA ..AAG.ATG. .ATTATTTTC ..GTT.CTGT TACATATA.. C.A(AAATGA AATT)*4 AA GATTCGATTT ---------- ---------- ---------- [1200]
A.in CC..A..T.A GT.C.TGA.- A..GTAT.GC ..GAAAATTT .T.TG..C.. CTG.TG.ACT T.TA-AGTAT GAAA.A---- A..TTTAAGG CATCG.T.TT A.AC..AGTT .G.TTAT.GT [1200]
B.pa CT..A..T.. ..A.GCC.G. -.CT...C.. ....--.... ......C.G. CA..TA..C. .G.A.GGTA. GG..AC---- --...C..AA (TCAAATTGA TTTATAATAA T)*28T.... [1200]

P.co TTGGTTTGTT GTTGCATTAT ATAGTGTTTT GTAAAAGCAC AAACAAGCAC ATCTTTATTC CCCAAGCCAA ATGGCCGGGA ACTAGATC-- TTTAAGCTAT -TATAAATCC AATATGAACA [1320]
G.p1 ---------- ---------- ---------- ---------- ---------- ---------- ---------- ---------- ---------- ---------- ---------- ---------- [1320]
G.p2 A.T.A..A.. AACAA.CAGA G.TAA...CC TG...TAG(A ATGCTATATA CAGCATTGTC A)*35 AATG CTATGAT--- ---------- ---------- ---------- ---------- [1320]
A.t1 ---------- ---------- ---------- ---------- ---------- ---------- ---------- ---------- ---------- ---------- ---------- ---------- [1320]
A.t2 AGATGTATGT ATATATATAT ATAATCCTTT ATAAAATAAA GTAGGCTCG) *2 ACATGC. GA.GTTT..G .G.CGTTTTT .A.GCCC.AT CAGG.CT.TA GCC.CCCA.T ..GTC----- [1320]
L.g1 ---------- ---------- ---------- ---------- ---------- ---------- ---------- ---------- ---------- ---------- ---------- ---------- [1320]
L.g2 TATTTAAAGC C)*22GCAC. ...TA..GG. .CCT.TTT.A ..C.TGCGGG GGT....AAG .AGCCCTGCC CA.T.TT.TC .GGCAC.--- ---------- ---------- ---------- [1320]
L.l1 CAACAA---- -------ATC TACAA.C--- ---(AAAACT A)*6.---.A .A..G--AGA TTT..----- ---------- ---------- ---------- ---------- ---------- [1320]
L.l2 CAACAA.--- --.AATGAT. TACTAA.AAG ..GCG..ATA GGTA.C--.A CA..G--.AA TTT.TT.TGC T..TTGTACT ...(TATACT ATTTATATA) *39TATGCTA TTTATATGGA [1320]
P.i1 ---------- ---------- ---------- ---------- ---------- ---------- ---------- ---------- ---------- ---------- ---------- ---------- [1320]
P.i2 ACCCAGCCCA T.ACT...TC G..A.ACC.C A.G.CTCGTA .T.GGTT.G. C..GG.GGC. AAATTTA-.. .CAA.TCAA( TTAAATTAAC AACTTATAGT GTG)*20TTA AATTAACAAC [1320]
C.az ---------- ---------- ---------- ---------- ---------- ---------- ---------- ---------- ---------- ---------- ---------- ---------- [1320]
C.ko ---------- ---------- ---------- ---------- ---------- ---------- ---------- ---------- ---------- ---------- ---------- ---------- [1320]
C.va ---------- ---------- ---------- ---------- ---------- ---------- ---------- ---------- ---------- ---------- ---------- ---------- [1320]
A.po ---------- ---------- ---------- ---------- ---------- ---------- ---------- ---------- ---------- ---------- ---------- ---------- [1320]
B.my ---------- ---------- ---------- ---------- ---------- ---------- ---------- ---------- ---------- ---------- ---------- ---------- [1320]
C.lu ---------- ---------- ---------- ---------- ---------- ---------- ---------- ---------- ---------- ---------- ---------- ---------- [1320]
A.in G.AA.GGTGA T.G.ATA... ..TCCA.AAA A.T.C.TTTG C.TA..TTTA .AA.A..... A...TTTAT. G..TA.ACT. TAA.C.GAAA ACC..AAA-- ---------- ---------- [1320]
B.pa ...A..CAGG CCC...CC.- ---------- ---------- ---------- ---------- ---------- ---------- ---------- ---------- ---------- ---------- [1320]

P.co ACTAAAAGCC CTGGCCTGTA GCTTTTTAGG T--ATTCTAC GGGCCCTAAA AAGACTAACA AACAGAATAT TTGC(ATATTAC A)*57 AT GCTAAAAATA TCGTAATATT TATAACACTA [1440]
G.p1 ---------- ---------- ---------- ---------- ---------- ---------- ---------- ---------- ---------- ---------- ---------- ---------- [1440]
G.p2 ---------- ---------- ---------- ---------- ---------- ---------- ---------- ---------- ---------- ---------- ---------- ---------- [1440]
A.t1 ---------- ---------- ---------- ---------- ---------- ---------- ---------- ---------- ---------- ---------- ---------- ---------- [1440]
A.t2 ---------- ---------- ---------- ---------- ---------- ---------- ---------- ---------- ---------- ---------- ---------- ---------- [1440]
L.g1 ---------- ---------- ---------- ---------- ---------- ---------- ---------- ---------- ---------- ---------- ---------- ---------- [1440]
L.g2 ---------- ---------- ---------- ---------- ---------- ---------- ---------- ---------- ---------- ---------- ---------- ---------- [1440]
L.l1 ---------- ---------- ---------- ---------- ---------- ---------- ---------- ---------- ---------- ---------- ---------- ---------- [1440]
L.l2 CAC------- ---------- ---------- ---------- ---------- ---------- ---------- ---------- ---------- ---------- ---------- ---------- [1440]
P.i1 ---------- ---------- ---------- ---------- ---------- ---------- ---------- ---------- ---------- ---------- ---------- ---------- [1440]
P.i2 C.AT.TG.GG TGTTAACAAC TACC.GG.AA GCTCAG.CGA ATAT------ ---------- ---------- ---------- ---------- ---------- ---------- ---------- [1440]
C.az ---------- ---------- ---------- ---------- ---------- ---------- ---------- ---------- ---------- ---------- ---------- ---------- [1440]
C.ko ---------- ---------- ---------- ---------- ---------- ---------- ---------- ---------- ---------- ---------- ---------- ---------- [1440]
C.va ---------- ---------- ---------- ---------- ---------- ---------- ---------- ---------- ---------- ---------- ---------- ---------- [1440]
A.po ---------- ---------- ---------- ---------- ---------- ---------- ---------- ---------- ---------- ---------- ---------- ---------- [1440]
B.my ---------- ---------- ---------- ---------- ---------- ---------- ---------- ---------- ---------- ---------- ---------- ---------- [1440]
C.lu ---------- ---------- ---------- ---------- ---------- ---------- ---------- ---------- ---------- ---------- ---------- ---------- [1440]
A.in ---------- ---------- ---------- ---------- ---------- ---------- ---------- ---------- ---------- ---------- ---------- ---------- [1440]
B.pa ---------- ---------- ---------- ---------- ---------- ---------- ---------- ---------- ---------- ---------- ---------- ---------- [1440]
```

```
P.co TTTATCAAAA TACTCAAATT TGTGGTGCCC AGGATATTTA GAACAC(TTT AATGT)*26  TTTAGCTT-- ---------- ---------- ---------- ---------- ----------  [1560]
G.p1 ---------- ---------- ---------- ---------- ---------- ---------- ---------- ---------- ---------- ---------- ---------- ----------  [1560]
G.p2 ---------- ---------- ---------- ---------- ---------- ---------- ---------- ---------- ---------- ---------- ---------- ----------  [1560]
A.t1 ---------- ---------- ---------- ---------- ---------- ---------- ---------- ---------- ---------- ---------- ---------- ----------  [1560]
A.t2 ---------- ---------- ---------- ---------- ---------- ---------- ---------- ---------- ---------- ---------- ---------- ----------  [1560]
L.g1 ---------- ---------- ---------- ---------- ---------- ---------- ---------- ---------- ---------- ---------- ---------- ----------  [1560]
L.g2 ---------- ---------- ---------- ---------- ---------- ---------- ---------- ---------- ---------- ---------- ---------- ----------  [1560]
L.l1 ---------- ---------- ---------- ---------- ---------- ---------- ---------- ---------- ---------- ---------- ---------- ----------  [1560]
L.l2 ---------- ---------- ---------- ---------- ---------- ---------- ---------- ---------- ---------- ---------- ---------- ----------  [1560]
P.i1 ---------- ---------- ---------- ---------- ---------- ---------- ---------- ---------- ---------- ---------- ---------- ----------  [1560]
P.i2 ---------- ---------- ---------- ---------- ---------- ---------- ---------- ---------- ---------- ---------- ---------- ----------  [1560]
C.az ---------- ---------- ---------- ---------- ---------- ---------- ---------- ---------- ---------- ---------- ---------- ----------  [1560]
C.ko ---------- ---------- ---------- ---------- ---------- ---------- ---------- ---------- ---------- ---------- ---------- ----------  [1560]
C.va ---------- ---------- ---------- ---------- ---------- ---------- ---------- ---------- ---------- ---------- ---------- ----------  [1560]
A.po ---------- ---------- ---------- ---------- ---------- ---------- ---------- ---------- ---------- ---------- ---------- ----------  [1560]
B.my ---------- ---------- ---------- ---------- ---------- ---------- ---------- ---------- ---------- ---------- ---------- ----------  [1560]
C.lu ---------- ---------- ---------- ---------- ---------- ---------- ---------- ---------- ---------- ---------- ---------- ----------  [1560]
A.in ---------- ---------- ---------- ---------- ---------- ---------- ---------- ---------- ---------- ---------- ---------- ----------  [1560]
B.pa ---------- ---------- ---------- ---------- ---------- ---------- ---------- ---------- ---------- ---------- ---------- ----------  [1560]
```
