# Supplementary material for: Mechanisms of gene rearrangement in 13 bothids based on comparison with a newly completed mitogenome of the threespot flounder, Grammatobothus polyophthalmus (Pleuronectiformes: Bothidae)
Source: BMC Genomics. 2019 Oct 30;20:792. doi: 10.1186/s12864-019-6128-9 (PMC6821024; doi:10.1186/s12864-019-6128-9)
Supplement: Supplementary file 4 — Additional file 4: Table S2. Primers used for fragment amplification of the G. polyophthalmus mitogenome. [file 12864_2019_6128_MOESM4_ESM.docx]

**Additional file 4: Table S2. Primers used for fragment amplification of the *G*. *polyophthalmus* mitogenome.**

| **Forward** | **Sequence*** **(5′-3′)** | **Reverse** | **Sequence (5′-3′)** |
| --- | --- | --- | --- |
| Z15 | ATTAAAGCATAACHCTGAAGATGTTAAGAT | F2671 | AGATAGAAACTGACCTGGAT |
| 16SAR | CGCCTGTTTATCAAAAACAT | 16SBR | CCGGTCTGAACTCAGATCACGT |
| ZILE | CTTGCCCTGGTTGTATGA | FCOI-70 | CCHACYATNCCDGCYCARGCMCCRAA |
| ZCOI-1295 | TNACHTTYTTYCCNCARCAYTTC | FLYS | CACCADTYTYYAGMTYAAAAG |
| ZATP-6310 | ACHTTYACNCCHACHACNCARCTNTC | FND4-865 | CCYATGTGVCYNACDGADGAGTADGC |
| Z10818 | TTYGAAGCAGCCGCMTGATACTGACAYTT | F13413 | TAGCTGCTACTCGGATTTGCACCAAGAGT |
| ZND6-210 | GCHARNGCNRCHGARTANGCAAA | FCYTB-95 | CCNARNARDGANCCRAARTTTCA |
| FCYTB-065 | CGCTAATGACCAACCTGCGAAAG | RND6-065 | GTCTTGGGTCTTGTGGCTGTTGCT |
| FND6-065 | GAGCGTCTATGTTCGCCAAGGT | RZ15-065 | CCACTCTTTACGCCGCTTTCTGT |

*Information of degenerate bases in the sequences as follows: H = A/C/T, Y=C/T, N=A/C/G/T, D=A /G /T, R=A/G, and M =A/C.
